# Editorial Comment from Dr Hamidi to Cranial nerve palsy caused by metastasis to the skull base in patients with castration‐resistant prostate cancer: Three case reports

**DOI:** 10.1002/iju5.12260

**Published:** 2021-02-12

**Authors:** Nurullah Hamidi

**Affiliations:** ^1^ Department of Urology Dr. Abdurrahman Yurtaslan Ankara Oncology Training and Research Hospital Ankara Turkey

I read with great interest Yasumizu *et al*.’s case report.^1^ The authors reported three castration‐resistant prostate cancer patients who developed cranial nerve palsy caused by skull base metastasis. Metastases were detected by magnetic resonance imaging (MRI), and external beam radiotherapy (EBRT) was planned for all patients. However, all patients died within a short time.

Skull base metastasis is a rare manifestation of advanced prostate cancer and its symptoms seriously worsen the quality of life of patients. Various cranial nerves may be involved. MRI is the first imaging modality due to limitations of bone scintigraphy and cranial computed tomography in symptomatic patients.^1^

Although the response to EBRT is quite good for symptom relief, early diagnosis is undoubtedly very important. Because, the prognosis of symptomatic patients with skull base metastasis is poor and many patients will die in a few months after symptoms are seen.^1^, ^2^ However, the answer to the question of which patients should be screened for cranial metastases is still unclear.

Another important point in these patients is symptoms relief. Because, symptoms of cranial nerve palsies negatively affect the quality of life. Corticosteroids will reduce edema around the cranial nerves and it will provide a significant improvement in patients' symptoms, especially during EBRT.

## Conflict of interest

The author declares no conflict of interest.

## References

[1] Yasumizu Y , Kosaka T , Hongo H , Mizuno R , Oya M . Cranial nerve palsy caused by metastasis to the skull base in patients with castration‐resistant prostate cancer: three case reports . IJU Case Rep. 2021; 4: 108–11. 10.1002/iju5.12255 PMC7924080 33718819

[2] McDermott RS , Anderson PR , Greenberg RE , Milestone BN , Hudes GR . Cranial nerve deficits in patients with metastatic prostate carcinoma: clinical features an treatment outcomes. Cancer 2004; 101: 1639–43. 15468187 10.1002/cncr.20553

